# UPF201 Archaeal Specific Family Members Reveal Structural Similarity to RNA-Binding Proteins but Low Likelihood for RNA-Binding Function

**DOI:** 10.1371/journal.pone.0003903

**Published:** 2008-12-11

**Authors:** Krishnamurthy N. Rao, Stephen K. Burley, Subramanyam Swaminathan

**Affiliations:** 1 Biology Department, Brookhaven National Laboratory, Upton, New York, United States of America; 2 Eli Lilly and Company, San Diego, California, United States of America; University of Queensland, Australia

## Abstract

We have determined X-ray crystal structures of four members of an archaeal specific family of proteins of unknown function (UPF0201; Pfam classification: DUF54) to advance our understanding of the genetic repertoire of archaea. Despite low pairwise amino acid sequence identities (10–40%) and the absence of conserved sequence motifs, the three-dimensional structures of these proteins are remarkably similar to one another. Their common polypeptide chain fold, encompassing a five-stranded antiparallel β-sheet and five α-helices, proved to be quite unexpectedly similar to that of the RRM-type RNA-binding domain of the ribosomal L5 protein, which is responsible for binding the 5S- rRNA. Structure-based sequence alignments enabled construction of a phylogenetic tree relating UPF0201 family members to L5 ribosomal proteins and other structurally similar RNA binding proteins, thereby expanding our understanding of the evolutionary purview of the RRM superfamily. Analyses of the surfaces of these newly determined UPF0201 structures suggest that they probably do not function as RNA binding proteins, and that this domain specific family of proteins has acquired a novel function in archaebacteria, which awaits experimental elucidation.

## Introduction

Understanding the origins of and evolutionary relationships among the three domains of life (archaea, eubacteria, and eukaryotes) constitutes one of the great challenges for post-genomic biology. The archaea remain the most enigmatic of the three [1]–[5]. In part, archaea are of interest, because they resemble eubacteria in some respects and eukaryotes in others [6]. They also hold considerable promise for the biotechnology industry [7]–[10]. Many archaeal organisms are thermophilic and some even survive at temperatures >100°C, and represent the only known strictly anaerobic methanogens on the planet [11]–[14]. Better known archaebacteria include *Methanococcus jannaschii*, *Sulfolobus solfataricus*, *Archaeoglobus fulgidus*, and *Methanobacterium thermoautotropicum*. These organisms are each members of the two major archaeal groups, namely crenarchaeota and euryarchaeota, defining all the basic molecular life machinery [5], [15], [16].

Following complete genome sequencing for the organisms listed above, ∼30% of the encoded ORFs were found to be archaeal specific [17]–[20]. Moreover, about a quarter of the archaeal genomes encode functionally uncharacterized proteins, most of which are common to other archaeal genomes [17]. UPF0201 family proteins constitute one such uncharacterized, archaeal specific protein family. Within the Pfam database, the UPF0201 proteins are classified under DUF54 entry (http://pfam.jouy.inra.fr/cgi-bin/getdesc?nameDUF54, accession number PF01877) and are related to conserved domain families COG1931 and COG1325 [21]. The DUF54 cluster includes 35 proteins (1–3 per organism), which are typically annotated as proteins of unknown function. In most cases, the Pfam domain spans most of the length of the predicted polypeptide chain. The two exceptions being a putative dephospho-CoA kinase (CoaE) from rice cluster I methanogen and a protein of unknown function (designated AF 1395) from *Archaeoglobus fulgidus*, wherein both Pfam domains map to the protein C-termini.

The New York SGX Research Center for Structural Genomics (NYSGXRC; www.nysgxrc.org) targeted four archaeal specific, UPF0201 family proteins for structural characterization and functional annotation, from among thermoacidophiles and hyperthermophiles (both methanogens), representing the two major archaeal phyla crenarchaeota and euryarchaeota [5], [15], [16]. Unexpectedly, the UPF0201 family member structures proved to be similar to those of the ribosomal L5 proteins, which are responsible for binding to 5S RNA. In addition to comparing and contrasting the four UPF0201 protein structures, we have used structure based sequence alignments to construct a phylogenetic tree that relates UPF0201 family members to L5 ribosomal subunits and other structurally similar RNA binding proteins, thereby extending the evolutionary purview of the RRM motif superfamily. Analyses of the surfaces of these newly determined UPF0201 structures suggest that they probably do not function as RNA binding proteins, and that this domain specific family of proteins has acquired a novel function in archaebacteria, which awaits experimental elucidation.

## Materials and Methods

### Gene cloning and protein production

Within the NYSGXRC, UPF0201 archaeal specific family proteins were assigned to target group 10077 (10077a: (Q58959) from *Methanococcus jannaschii*; 10077b: (Q97Z89) from *Sulfolobus solfataricus*; 10077c: (Q9UXC9) from *Sulfolobus solfataricus* P2; 10077d (O27966) and 10077e: (O28876) from *Archaeoglobus fulgidus*; and 10077h: (O26533) from *Methanobacterium thermoautotrophicum*). Genes encoding these proteins were amplified from genomic DNA using the polymerase chain reaction. Gene cloning and protein expression/purification utilized previously published NYSGXRC protocols, which are described in detail in PepcDB (www.pepcdb.pdb.org). Mass spectrometry analyses documented that none of the purified proteins had undergone degradation or post-translational modification (data not shown).

### Crystallization and diffraction data collection

Crystallization screening and further optimization *via* sitting drop vapor diffusion with Se-Met protein samples yielded optimal conditions for each UPF0201 target as follows: 10077a-10 mM HEPES pH 7.5, 0.2 M ammonium acetate, 25% PEG 3350; 10077b-10 mM sodium citrate pH 5.5, 20% (v/v) isopropanol, 20% PEG 4K; 10077c-3.5 M sodium formate pH 7.0; 10077d-10 mM HEPES pH 7.0, 5% tascimate pH 7.0, 10% PEGMME 5K. Crystals were flash frozen by direct immersion in liquid nitrogen following addition of 15–20% glycerol as a cryo-protectant. All X-ray diffraction data were recorded using beamline X12C at the National Synchrotron Light Source, Brookhaven National Laboratory. Data were processed and scaled using HKL2000 [22]. See Table 1 for a summary of crystallographic data statistics.

**Table 1:** Data Collection and Refinement Statistics.

Target ID	10077a	10077b	10077c	10077d
X-ray Wavelength (Å)	0.9792	0.9797	0.9795	0.9796
Space Group	P1	P2_1_2_1_2_1_	R32	P2_1_2_1_2_1_
Unit Cell (Å)	a = 46.5	a = 44.7	a = 127.5	a = 38.4
	b = 50.2	b = 66.3	b = 127.5	b = 156.3
	c = 73.8	c = 124.7	c = 61.6	c = 174.7
	α = 70.3,β = 72.6,γ = 84.3°			
Resolution Limit (Å)	50-2.2	50-2.3	50-2.5	50-3.0
Outer shell resolution (Å)	2.28-2.20	2.38-2.30	2.5-2.59	3.12-3.0
No. of unique reflections	30128	16881	6695	21971
Redundancy	7.2 (5.3)	14.2 (14.0)	9.2 (8.2)	6.6 (6.1)
Rmerge (%)^a^ ^1^	7.0 (27.8)	5.8(23.1)	5.5 (35.4)	8.4(44.6)
Overall completeness (%)	98.9 (90.9)	98.9 (97.9)	99.9(99.5)	99.5 (99.1)
〈I/σ(I)〉	14.6 (3.2)	16.4(2.4)	14.1(3.5)	8.7 (2.3)
**Refinement Statistics**				
Resolution range (Å)	47-2.2	50-2.4	50-2.6	50-3.0
No. of reflections	29198	13975	5642	21209
R-factor^b^	0.249	0.251	0.245	0.262
*R*-free^c^	0.299	0.298	0.303	0.309
No. of protein atoms	3843	2117	1100	4235
No. of water molecules	83	52	17	44
**Geometry**				
Bond length r.m.s.d.s (Å)	0.007	0.015	0.007	0.008
Bond angles r.m.s.d.s (°)	1.3	1.3	1.2	1.3
**Ramachandran Analysis**				
Residues in (%)				
core region	85.2	91.8	88.5	90.1
additionally allowed	13.7	8.2	11.5	9.5
generously allowed	0.7	0.0	0.0	0.4
disallowed	0.7	0.0	0.0	0.0

1 The values corresponding to the outermost shell are given within parentheses.

a *R*-merge = Σ_h_Σ_i_|*I*_h,i_−〈*I*_h_〉|/Σ_h_Σ *I*_h,i_ where 〈*I*_h_〉 is the mean intensity of symmetry-related reflections, *I*_h,i_.

b *R*-factor = Σ||*F_o_*|−|*F_c_*||/Σ|*F_o_*| where *F_o_* and *F_c_* are the observed and calculated structure factor amplitudes, respectively.

c *R*-free is calculated for about 2% of the data withheld from refinement.

### Structure determination

All structures were determined independently *via* single wavelength anomalous dispersion (SAD) with Se-Met crystals. In each case, SAD data collection at an X-ray wavelength corresponding to the crystal Se emission line sufficed for determining the Se atom substructure with SHELXD [23]. For 10077a, crystals were obtained in a triclinic space group with 4 molecules in the asymmetric unit, and the structure could only be determined after combining two full-sphere SAD data sets recorded from two crystals. Initial phases were obtained with SHARP [24], and further improved *via* density modification using DM [25]. In all cases, about 70% of the polypeptide chain was built automatically by *ARP*/*wARP* [26] except in the case of 10077d where the data extended to 3 Å only. Subsequent model building was performed manually using *O* [27]. Structure refinement was performed with simulated annealing followed by Powell energy minimization [28]. The refined atomic model was evaluated using the RCSB *AUTODEP* deposit tool (www.pdb.org). Final refinement statistics are given in Table 1.

### Computational tools for structure analysis

1) Secondary structural elements, hydrogen bonds, solvent accessible surface area, buried residues, and folding free energy were calculated using VADAR [29]. 2) Ionic interactions (salt bridges) and cation-pi interactions were calculated using PIC [30]. 3) Secondary structure attribution of residues and hydrogen bonds were calculated using DSSP [31]. 4) Contact and potential energies were calculated with PSQS [32]. 5) To calculate geometry of the probable protease, glycolysis pathway enzyme, or metal binding motif residues PAR-3D (http://sunserver.cdfd.org.in:8080/protease/PAR_3D/access.html) was used, though none was identified [33]. 6) Putative RNA binding residues were identified using BindN (http://bioinfo.ggc.org/bindn/), RNAbindR (http://bindr.gdcb.iastate.edu/RNABindR/), and KYG (http://yayoi.kansai.jaea.go.jp/qbg/kyg/index.php) [34]–[36]. 7) Conserved residues were mapped onto the structure using ConSurf [37]. 8) For phylogenetic analysis the structure based multiple sequence alignment and the resulting tree was constructed using 3Dcoffee choice from the T-coffee package (http://www.tcoffee.org/) [38].

## Results and Discussion

### Crystallization outcomes

Cloning, expression, and purification of various truncated and tagged forms of the 10077 targets were performed in the context of the standard NYSGXRC approach to structure determination. For 10077a from *M. jannaschii*, full-length constructs with either N- or C-terminal His_6_ affinity tags failed to yield crystals. C-terminal truncation of 30 amino acids yielded diffraction quality crystals and a structure. For 10077b and 10077c from *S. solfataricus* and *S. solfataricus* P2, respectively, full-length constructs bearing C-terminal His_6_ tags yielded crystals and structures. For 10077d from *A. fulgidus*, the N-terminal His_6_ tagged full length protein gave crystals and a structure, whereas the C-terminal His_6_ tagged version yielded neither. For 10077h from *M. thermoautotrophicum*, neither N- nor C-terminal His_6_ tagged versions of the full length protein yielded crystals. In none of the three X-ray structures of full-length UPF0201 proteins was electron density corresponding to the 15–20 C-terminal residues observed. Both the pI values and the protein hydropathy scores for successfully crystallized UPF0201 proteins fall within ranges most commonly observed for successful crystallization of another thermophile, *Thermotoga maritima* by the Joint Center for Structural Genomics [39].

### Overall structure of the UPF0201 protomer

**Figure 1:**
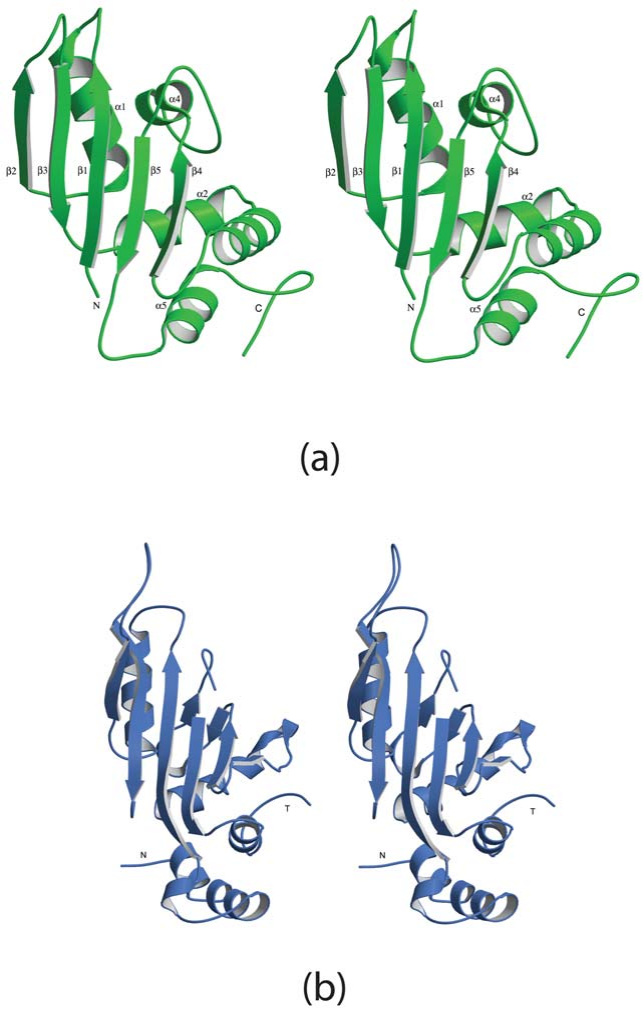
Ribbons stereodrawing of representative a) UPF0201 (10077a, upper) and b) L5 (BstL5, lower) proteins. Prepared using Molscript [75].

The UPF0201 family proteins occur as a single globular α/β domain (Figure 1a) with approximate dimensions of 55×35×35 Å^3^. Despite very low sequence similarity among the UPF0201 proteins (pairwise amino acid identities = 15–35%) the overall polypeptide chain fold is conserved (Cα atom pairwise root-mean-square-deviations or r.m.s.d.s = 1.5–2.9 Å (for about 110–120 Cα pairs). The protomeric structure consists of a five-stranded, anti-parallel β-sheet, five α helices, which are located on one face of the β-sheet, and three loops connecting helices and strands. Secondary structural elements occur in the following order: β1–α1–β2–β3–α2–α3–β4–α4–β5–α5 (Figure 1a). The order of strands in the β-sheet is β2–β3–β1–β5–β4. The loop connecting β2 and β3 protrudes somewhat from the globular domain, and the electron density corresponding to this region is poorly defined in the 10077b and 10077d structures. In contrast, the loop connecting β4 and β5 is well defined in all four structures. The polypeptide chains of 10077a and 10077b extend beyond the C-terminal helix, α5, for about 20 residues, and form a type IV turn followed by random coil.

### Structure Comparison

An automated DALI search for structural homologs of the four UPF0201 family members (10077a, 10077b, 10077c, 10077d) in the Protein Data Bank (PDB; www.pdb.org) [40] revealed structural similarity with a number of single domain α/β RNA-binding proteins, with the majority being ribosomal L5 proteins (Figure 1b). Chain A of 10077a most closely resembles various extant structures of L5, including those from *Bacillus sterothermophilus* [41] (BstL5: PDB Code 1IQ4, Z-score = 9.3, Sequence Identity = 14%, r.m.s.d. = 2.6 Å for 113 equivalent Cα pairs), *Thermus thermophilus* [42] (TtL5: PDB Code 1MJI, Z-score = 8.7, Sequence Identity = 14%, r.m.s.d. = 2.8 Å for 113 equivalent Cα pairs), *Haloarcula marismortui* [43], [44] (HmaL5: PDB Code 1JJ2, Z-score = 7.7, Sequence Identity = 16%, r.m.s.d. = 2.4 Å for 96 equivalent Cα pairs), and *E. coli* [46], [47] (EcolL5: PDB ID 2AWB, Z-score = 5.6, Sequence Identity = 8%, r.m.s.d. = 3.6 Å for 108 equivalent Cα pairs).

Notwithstanding low pairwise amino acid sequence identities (8–16%) the core regions of the UPF0201 and L5 structures are quite similar. Substantive differences are largely confined to the N- and C-termini and various loop regions (Figure 1a and 1b). Both eubacterial and archaeal L5 ribosomal subunits are about ∼180 residues in length and typically share ∼55% sequence identity, with pairwise Cα r.m.s.d.s of 3.5 Å among structurally characterized L5 proteins [42]. Unlike the UPF0201 family members and the archaeal HmaL5 protein, eubacterial L5 subfamily members lack α1 and possess shorter β2–β3 and β4–β5 segments. All L5 proteins lack the extended C-terminus and the region corresponding to residues 80–90 in UPF0201 family members, which forms helix α3. Thus, the 10077 NYSGXRC targets are almost certainly not ribosomal L5 subunits *per se*.

Next we examine the structural relationships between UPF0201 family members and other entries in the PDB. Not surprising given the similarity of the UPF0201 family members to ribosomal L5, Chain A of 10077a resembles the U1A RNP from human (U1A; PDB ID 1OIA, Z-score = 1.7, Sequence Identity = 15%, r.m.s.d. = 3.4 Å for 67 equivalent Cα pairs). U1A is an RNA binding protein comprising the RNA recognition motif (or RRM), which forms part of the ribonucleoprotein complex involved in the excision of introns [48], [49]. Following is the comparison of 10077a with other RNA binding proteins; for U2 snRNP protein U2B″ [50] (PDB code 1A9N), Z-score = 1.8, Sequence Identity = 11%, r.m.s.d. = 4.7 Å for 63 equivalent Cα pairs; for YxiN protein [51] (PDB code 2G0C), Z-score = 1.6, Sequence Identity = 10%, r.m.s.d. = 2.8 Å for 50 equivalent Cα pairs; for alternative splicing factor Sxl [52](PDB code 1B7F), Z-score = 2.3, Sequence Identity = 11%, r.m.s.d. = 2.9 Å for 62 equivalent Cα pairs; for PAB [53](PDB code 1CVJ), Z-score = 2.4, Sequence Identity = 10%, r.m.s.d. = 3.3 Å for 63 equivalent Cα pairs; for pre-rRNA packaging protein nucleolin RBD12 [54] (PDB code 1FJE), Z-score = 1.8, Sequence Identity = 5%, r.m.s.d. = 3.9 Å for 62 equivalent Cα pairs; for translation regulatory protein HuD [55] (PDB code 1FXL), Z-score = 3.0, Sequence Identity = 6%, r.m.s.d. = 3.1 Å for 64 equivalent Cα pairs.

Among other UPF0201 proteins homologs in the PDB 10077c was identified by DALI as similar to NikR from *H. pylori* [56] (HpNikR: PDB ID 2CAJ, Z-score = 4.2, Sequence Identity = 4%, r.m.s.d. = 2.9 Å for 68 equivalent Cα pairs), and 10077d most closely resembles NIKR from *E.coli* [57] (EcNikR: PDB ID 2BJ1, Z-score = 2.2, Sequence Identity = 6%, r.m.s.d. = 3.8 Å for 66 equivalent Cα pairs). The NikRs have been characterized as nickel responsive gene regulators in eubacteria. Superposition of the *H. pylori* and *E. coli* NikR proteins (PDB IDs 2CAJ and 2BJ1, respectively) onto our four UPF0201 protein structures revealed structural similarity only within the NikR C-terminal tetramerization domain (TD). Given that the DALI overlays involve only part of the structurally-conserved target 10077 globular domain and that these UPF0201 proteins lack conserved Ni^++^ ion binding residues, we believe it extremely unlikely that the UPF0201 family member proteins contribute to gene regulation in response to metal ions in archaebacteria.

No other statistically significant hits were obtained from our DALI search of the PDB. We conclude, therefore, that the UPF0201 family members have proven quite unexpectedly, from the standpoint of amino acid sequence relationships alone, to be members of the RRM superfamily [58].

### Functional Annotation

#### UPF0201/DUF54 Sequence-Sequence Relationships

**Figure 2:**
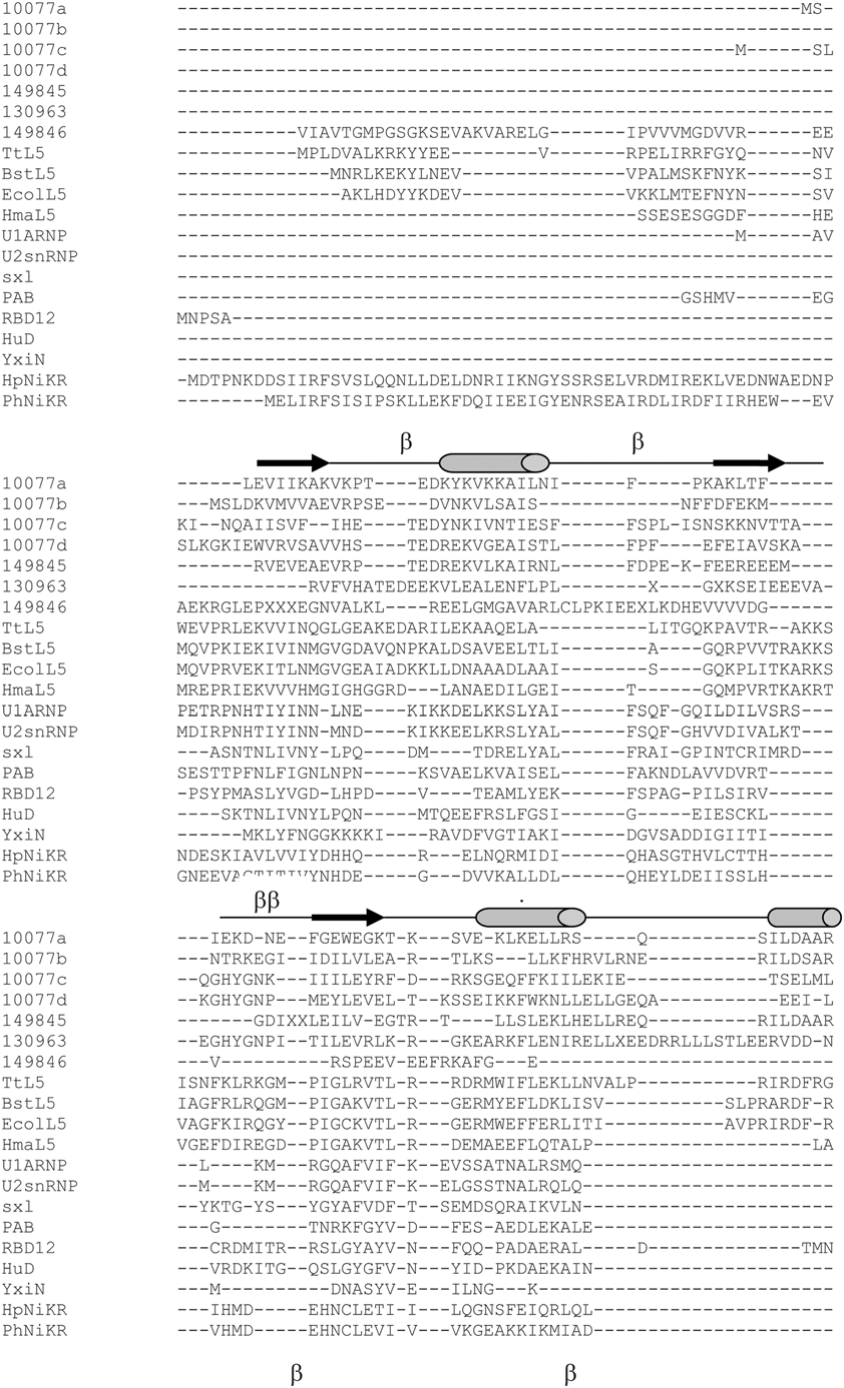
Sequence alignment of UPF0201 proteins (that include known structures and consensus sequences from 3 SYSTER families), L5 and non-L5 RRM containing proteins and NiKR. The secondary structural elements corresponding to 10077a (2PZZ) is shown at the top of the alignment with arrow marks and cylinders representing β strands and α helices. The turns are marked with the letter ‘β’.

Pairwise sequence identities among the structures we report herein range between 15–22%, with exception of 10077a and 10077b, which are 35% identical. The entire family of archaeal specific DUF54 (UPF0201) domains can be further classified into three sequence based SYSTERS protein families [59]. The SYSTERS protein family database provides information regarding the domain architecture of a protein and helps identify differences in domain composition within a protein family. For DUF54 (UPF0201), SYSTERS identified three subfamilies, including N149845 (10 non-redundant sequences, MW∼13 kDa), N149846 (16 non-redundant sequences, MW∼17 kDa) and N130963 (12 non-redundant sequences, MW∼15 kDa). Pairwise amino acid sequence identities are <25% among most of these proteins. Our four UPF0201 structures represent subfamilies N149845 (10077a, b) and N130963 (10077c, d). Figure 2 demonstrates that no residues are absolutely conserved among our four UPF0201 structures, and that there is minimal sequence conservation across the entire archaeal specific family of UPF0201 proteins. Notwithstanding these findings, the results of threading analyses suggest that the entire UPF0201/DUF54 family of archaeal specific proteins share the same overall RRM-type polypeptide chain fold.

### Sequence/Phylogenetic Analyses

**Figure 3:**
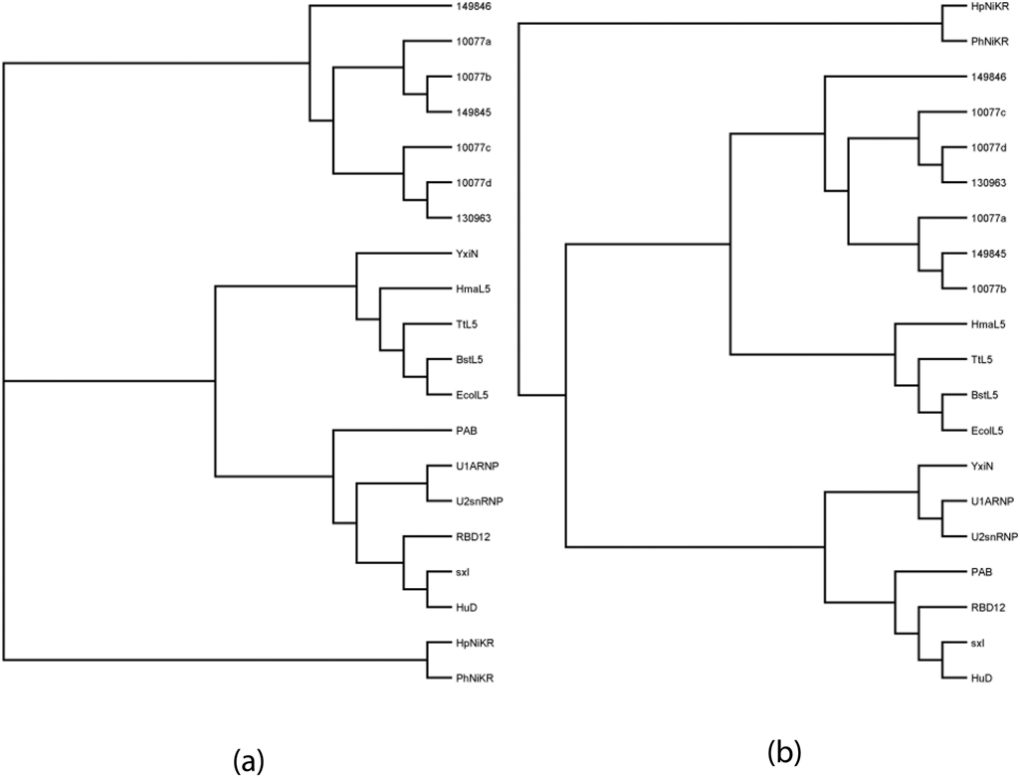
Differences in phylogenetic trees constructed from a) sequence based and b) structure based multiple sequence alignment. The sequences and structures of UPF0201 proteins and both L5 and non-L5 RRM homologs are included in the alignment.

Having demonstrated for the first time that the UPF0201 proteins are structurally similar to the RRM type RNA binding proteins, we sought to further investigate possible evolutionary relationships by comparing the sequences of all known UPF0201 proteins and structurally characterized L5/RRM proteins, for which accurate sequence alignments could be generated by identifying equivalent Cα atoms in structure-structure alignments. Use of structure-based alignments overcomes some of the errors that are inevitably introduced by attempting to align amino acid sequences directly when identities drop significantly below 20–25%. While the structural divergence exponentially decreases as the sequence similarity increases, the same is not true when then the sequence similarity is below 25% or so. Moreover, tertiary structures tend to be more conserved in evolution and retain the functional properties than sequences [60], [61]. Accordingly, the structure based phylogenetic tree is more informative than that based on sequence (Figure 3a and 3b). The structure based alignment can be produced in many different ways and we used 3DCoffee for structure based alignment using the coordinates of experimental models available in the PDB [38].

Figure 3b illustrates the results of performing structure-based sequence alignments for the UPF0201, L5, and non-L5 RRM proteins. As expected, the NiKR and RRM containing proteins (UPF0201 proteins, L5 proteins, non-L5 RRM type proteins) first divide into two branches reflecting their distant relationship. Further, the RRM containing proteins are divided into non-L5 RRM type proteins and L5 proteins. Under the non-L5 RRM proteins group the proteins mapped to separate branch reflect their unique function. The UPF0201 proteins segregate along with the L5 proteins and then map to sub groups according to their SYSTERS family classification scheme. Using structure based alignment the UPF0201 proteins could be classified under RRM containing proteins whereas in the sequence based tree they were placed in a separate branch. Presumably, due to very low sequence similarity the relationship of UPF0201 proteins with RRM containing proteins could only be established based on the structure. Within the UPF0201 family, the 3 SYSTERS families divide into 3 branches, the SYSTER families N149845 (10077a and b) and 130963 (10077c and d) segregate into one and then divide while SYSTER family N149846 is placed separately. Within the L5 family, proteins from the bacterial and archaeal domains map to separate branches. We suggest that the UPF0201 proteins and L5 and non L5 RRM type proteins originated from a common ancestral RRM-containing protein. We are able to show that proteins with no sequence homology but having close structural homology can be classified to the same group and further they can be classified into sub groups based on their functional similarity.

### Surface Analyses

**Figure 4:**
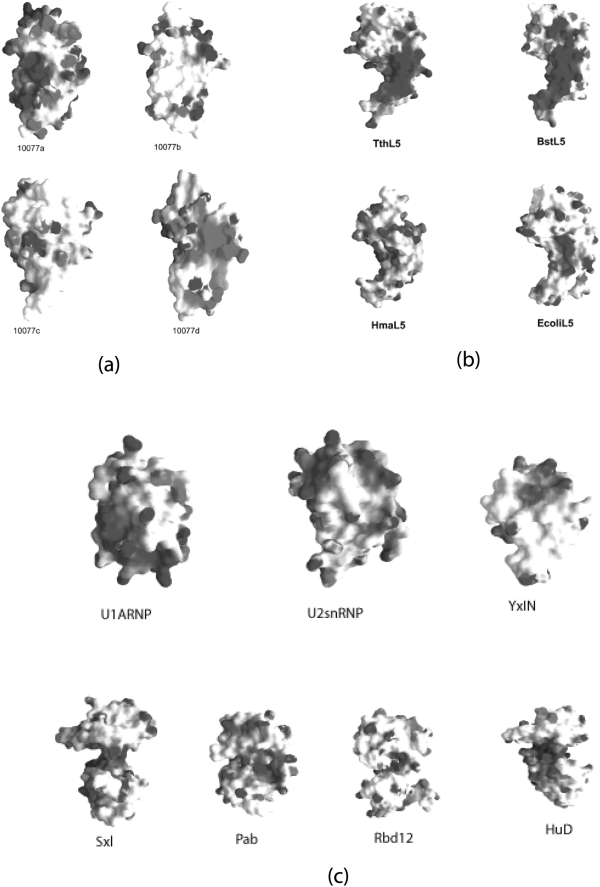
Electrostatic potential surfaces of a) UPF0201 proteins and selected b) L5 (PDB Code: 1IQ4 (BstL5), 1MJI (TtL5), 1JJ2 (HmaL5) and 2AWB (EcolL5) and c) non-L5 (PDB Code: 1OIA (U1ARNP), 2G0C (YxIN)) RRM homologs. Color-coding denotes calculated electrostatic potential (red: <−10 kT; blue: >+10 kT) and underlying residue conservation as acidic (red) and basic regions (blue).

**Figure 5:**
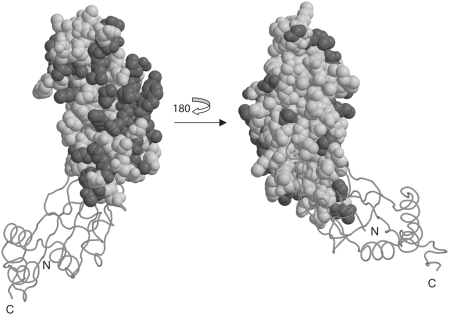
Based on multiple sequence alignment of UPF0201 proteins evolutionarily conserved (yellow) and most variable residues (red) were identified and mapped to three-dimensional structure of 10077a. The RNA binding surface (in L5) (left) and the opposite surface (right; 180 rotated from left one) are shown. The other monomer of the dimer is depicted as Cα trace in green.

Figure 4a–c illustrates the solvent-accessible surfaces of our four structures together with those of representative L5 and non-L5 RRM proteins, color coded for calculated electrostatic potential and underlying residue conservation. The surface representations of known L5 from archaea and bacteria and the U1A RRM from human and RNA binding YxiN protein of *Bacillus subtilis* both demonstrate conservation of basic and hydrophobic residues on the relatively flat RNA-binding surface corresponding to the exposed face of the five-stranded, anti-parallel β-sheet. 10077a–d do not share these properties. 10077a does display positive electrostatic potential feature in the vicinity of the open β-sheet face. In contrast, 10077d displays a cluster of negatively charged residues at the same site. The surfaces of 10077b and 10077c are electrostatically neutral throughout, including the site of rRNA binding to L5. We used three web servers, RNAbindR (http://bindr.gdcb.iastate.edu/RNABindR/), bindN (http://bioinfo.ggc.org/bindn/), and KYG (http://yayoi.kansai.jaea.go.jp/qbg/kyg/index.php), to identify putative RNA-binding residues for the UPF0201 proteins. Residues commonly identified by all the servers were mapped onto three-dimensional structures of UPF0201 proteins. Most of the putative RNA-binding residues, including Lys and Arg did not correspond to the known RNA-binding surface of the RRM. In fact, in all four UPF0201 proteins examined residues predicted to be involved in RNA binding are not conserved. Moreover, Figure 5 demonstrates that the least conserved residues (or most variable) occur on the exposed surface of the planar β-sheet where 5S RNA binds to the L5 proteins.

We, therefore, propose that the archaeal specific UPF0201 proteins do not represent a family of RNA binding proteins. Given that the overall shape of the molecular surface and calculated electrostatic potential vary among UPF0201 proteins and there are few absolutely conserved residues apparent in Fig. 2 and 5 it is formally possible that members of the DUF54 Pfam family possess different biological functions. For DUF62 Pfam family, we recently reported that function does vary among members [62]. Examination of surface conservation among 10077a–d revealed well-defined clusters of surface residues (Figure 5), including Val10, Thr13, Glu14, Asp15, Lys18, Val19, Ala22, Asn25, Ile63, Asp65, Ala67, Arg68, Lys86, Gln87, Ala89, Asn95, Ile104, Pro125, Thr127, Gly130 (using 10077a residue numbering). Intriguingly, the conserved patches could be mapped to the same space in three-dimension in all four structures. The conserved residues map to form a continuous patch on the backside of the β-sheet plane, the side comprising the opposite edge of the rRNA binding L5 surface.

### Thermal stability analysis of proteins

Thermostable proteins provide us means to understand the molecular basis for stability and to engineer more such proteins [63]–[67]. Since all the four proteins (10077a–d) involved in this study belong to thermostable class of proteins, we analyzed the probable reasons for thermal stability using their structures along with a few other structures (1IQ4, 1MJI, 1JJ2 and 2AWB) available in the Protein data Bank (Table 2). Even though the following analysis involves a small sample set, it has from mesophile to hyperthermophile proteins. Analysis of these structures indicated clear correlation of the factors such as hydrogen bonds, accessible surface area, density of salt bridges and compactness. Thermophiles and hyperthermophiles have twice the number of ionic interactions (salt bridges) and cation-pi interactions compared to mesophile, a feature commonly observed in other thermophiles [64], [68], [69]. Further, in our analysis several energetically favorable cation-pi interactions could be observed among thermophiles and hyperthermophiles while only a very few such type of interactions could be found in mesophile. Ionic and cation-pi interactions together form on an average of 18 bonds per protein chain of thermophiles compared to 9 bonds per chain for the mesophile. A clear trend could be observed with respect to number of hydrogen bonds and the number of residues in the secondary structure. Both these parameters were found to increase while going from mesophile to thermophiles to hyperthermophiles, which is in agreement with previously reported trend based on large-scale data analysis [69]–[72]. The latter factor further agrees with the fact that as thermophilicity increases the protein chains tend to be shorter and contained shorter loops than their mesophilic homologs, which is also consistent with the previous studies on large scale studies [70].. Another parameter we analyzed for the thermal stability is stabilization energy, which includes burial, local and contact energy [32]. Burial component of energy showed clear trend to increase from mesophile to thermophile to hyperthermophile while the contact potential found to be especially strong (mean difference = −0.0538). Such a trend is previously reported in the context of thermal stability of proteins from *Thermotoga maritima* genome [72], [74]. Thermophilic proteins have significantly lower relative accessible surface area (ASA) and avoid access to hot solvent regions in the cell and thus become more compact [72]. Specifically, we find that thermophiles display higher ASA to total volume ratio (0.55) compared to that of mesophiles (0.40). A few violations in Table 2 observed among mesophiles may be attributed to the low resolution (2AWB, 3.5 Å) of the structure included for thermal stability analysis. Though it is generally believed that disulfide bridges are important for thermostability none was observed in our small sample set [73]. We find that in our case, the lack of disulfide bonds is compensated by large number of ionic interaction helping in the stability of these proteins.

**Table 2:** Comparison of features of thermal stability between hyperthermophiles/thermophiles and mesophile.

Parameter	I	II	III	IV	V	VI
Protein Name (temp °C)						
110077a (85)	133/17630	7603/32	81(65)/65	15/4	−124.94/35.53	−0.062/−0.268
110077b (80)	131/17527	7220/32	75(70)/63	12/3	−116.49/40.42	−0.073/−0.214
110077c (83)	137/18601	7713/27	81(75)/70	16/2	−121.01/40.73	−0.079/−0.300
210077d (72)	133/17215	7370/31	78(75)/75	16/3	−113.76/38.52	−0.097/−0.434
21IQ4 (65)	179/23653	10472/39	72(64)/58	13/1	−163.78/36.52	−0.067/−0.256
21MJI (65)	178/24827	10651/38	69(67)/53	16/3	−158.01/29.08	−0.054/−0.174
31JJ2 (37)	140/18932	9612/20	65(60)/51	9/1	−103.45/47.40	−0.008/−0.154
32AWB (37)	178/24104	11210/15	61(61)/23	17/6	−107.97/−	−0.011/0.070

I - Total no of residues/Total volume in Å^3^.

II - Total accessible surface area (ASA, Å^2^)/No. of residues that are 95% buried.

III - % of residues in hydrogen bonds (total no of hydrogen bonds per 100 residues) / % of residues in secondary structure.

IV - No. of salt bridges/ No.of cation-pi interactions.

V - Total folding energy/ Protein instability index.

VI - Contact energy/ total potential energy.

1 Hyperthermophile. ^2^thermophile, and ^3^mesophile.

### Quaternary Structure

**Figure 6:**
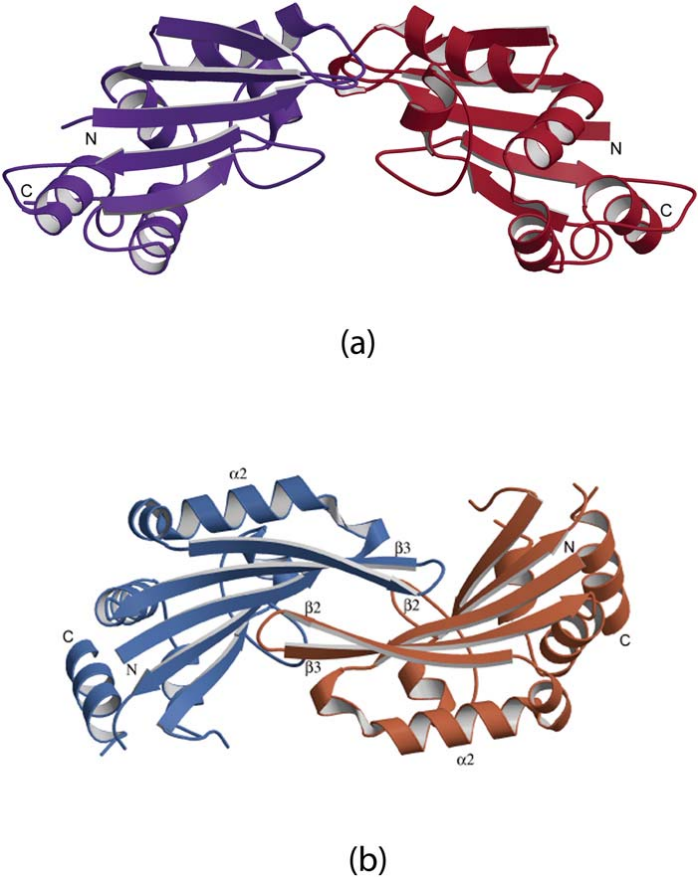
Ribbon diagram of UPF0201 proteins biological assemblies (dimers) a) 10077a, and b) 10077c. Each monomer is shown in different color.

Analytical gel filtration experiments, though only a rough estimate of mass, documented that proteins 10077a–d exist as dimers in solution and agree with the crystallographic results as discussed below. The crystallographic asymmetric units contain one protomer in 10077c, two in 10077b and four protomers in both 10077a and 10077d. The proteins distinctly form two types of biological assemblies as revealed by the analysis of the protein interfaces at PISA site http://www.ebi.ac.uk/msd-srv/prot_int/pistart.html. While 10077a, 10077b and 10077d all form one type of dimer (typeI, Figure 6a), 10077c forms altogether different dimer via crystallographic symmetry (typeII, Figure 6b). The interface surface area for type I dimers in 10077a, 10077b and 10077d are 672, 563 and 611 Å^2^, respectively. For type II dimer in 10077c, the interface surface area is larger and equal to 982 Å^2^. In type I the turn connecting β1 and helix α2 (residues Thr13-Asp15) assemble closely to form a two stranded β-sheet. At the type II interface the turn connecting -strand β1 and helix α2 interacts with the turn that connects helix α4 and β5 (residues Gly115-Asp117). In the structures that form type I assembly the latter turn points away from the interface or is disordered while the former turn has lengthier side chains causing short contacts and thereby destabilizing the interface. Majority of the interactions seen at the type II assembly interface are due to exchange of strands β2, β3, and the turn connecting them (residues Gly25-Asp50) between the two monomers. Moreover, this part of the structure in 10077c is about 5 residues longer compared to the other three. The interactions involve a large number of residues, which include Ser9, His13, Glu14, Thr15, Glu16, Asp17, His46, Asn49, Glu55, Asp116 and Gly117 of 10077c. Interestingly, structure alignment shows residues (Thr13, Glu14, Asp15, and Lys18 of 10077a) near the interface of type I assembly are strictly conserved while those seen at the type II assembly are not. Despite that the type II assembly involves large number of interactions and presumably more stable than the type I, such an assembly is seen only in one among the four structures reported here. Overall from this analysis we observe that protein-protein assembly chosen by the proteins may depend on the nature of the amino acid found at the interface since they can make necessary interactions leading to stability of the assembly.

### Conclusions

We have described determination of the structures of four UPF0201 proteins from three distinct archaebacteria. With these data, we have provided the first structural information regarding members of the UPF0201/DUF54 family. We have further documented that all members of this archaeal specific protein family share a common polypeptide chain fold, which is evolutionarily related to the RRM motif found in the ribosomal L5 proteins and many other RNA-binding proteins. Further structural characterization of the UPF0201/DUF54 family, either by molecular replacement or homology modeling, will be enabled by the structures of 10077a–d. Moreover, structure-structure comparisons have demonstrated that it is highly unlikely that these proteins share a common function with *bona fide* RNA-binding RRM proteins. The structures will, however, provide a rational basis with which to design experiments intended to establish the functional properties of UPF0201/DUF54 family members.

Atomic coordinates and structure factor amplitudes have been deposited into the Protein Data Bank with the following PDB IDs: 10077a-2PZZ, 10077b-2NRQ, 10077c-2NWU, and 10077d-2OGK,
